# Psychometric evaluation of the Disability Wellbeing Index (DWI) against the Adult Social Care Outcomes Toolkit (ASCOT) among people with disability

**DOI:** 10.1007/s11136-026-04406-6

**Published:** 2026-09-27

**Authors:** Gozde Aydin, Samia Badji, Dennis Petrie, Anthony Harris, Gang Chen

**Affiliations:** 1 Centre for Health Economics, Monash University, Melbourne, VIC Australia; 2 Centre for Health Policy, The University of Melbourne, Melbourne, VIC Australia

**Keywords:** DWI, ASCOT, Disability, Wellbeing, Social care, Construct validity

## Abstract

**Purpose:**

The Disability Wellbeing Index (DWI) is a preference-based wellbeing measure developed among people with disabilities. This study examined the psychometric properties of the DWI in both its Standard and Easy English versions and compared them with two Adult Social Care Outcomes Toolkit (ASCOT) instruments, the ASCOT-SCT4 and ASCOT-ER.

**Methods:**

We conducted an online survey among National Disability Insurance Scheme (NDIS) participants aged ≥ 15 years in 2024. DWI was scored using an Australian preference-based algorithm, and ASCOT was scored using UK preference weights. Convergent validity was assessed using Spearman’s correlations between total utility scores and corresponding domain items. We examined underlying constructs between DWI and ASCOT using exploratory factor analyses (EFAs), and known-groups validity using regression analyses comparing scores across groups based on self-rated health, self-advocacy, participation in meaningful leisure activities, and autonomy in decision-making.

**Results:**

A total of 1871 participants were studied; among them, 39% completed the Easy English/Read version, and 30% were aged 15–24 years. Correlations between DWI and ASCOT were moderate for young people (ρ = 0.62, Easy English/Read; ρ = 0.66, Standard) and strong for adults (ρ = 0.74, Easy English/Read; ρ = 0.75, Standard). Convergence was strongest for safety items, whereas convergence was weaker for dignity items. EFA indicated partial overlap in underlying constructs. Known-groups validity was supported for both measures.

**Conclusions:**

The findings support the construct validity of DWI. DWI and ASCOT focus on different but overlapping aspects. The DWI places a stronger emphasis on general wellbeing, while the ASCOT focuses on social care related quality of life.

**Supplementary Information:**

The online version contains supplementary material available at https://doi.org/10.1007/s11136-026-04406-6.

## Introduction

Approximately one in six people worldwide lives with a disability [1]. Across countries and contexts, people with disability experience persistent inequities in health [2], social participation [3], economic opportunity [4] and overall wellbeing [5]. In response to growing international calls for greater autonomy, inclusion, and participation, governments have increasingly invested in disability support systems and services [6]. As investment in disability services grows globally [7–9], rigorous evaluation is increasingly critical [10]. Evaluation tools must capture not only health-related improvements but also the broader dimensions of wellbeing valued by people with disability, such as dignity, safety, self-care and social inclusion [11–13].

Preference-based measures support evaluations by incorporating what matters to the population [14]. They can inform funding and resource allocation by identifying supports that deliver the greatest value for money. However, widely used generic instruments such as EQ-5D [15] and AQoL-8D [16] were designed for the general population and focus primarily on health status (with EQ-5D primarily focusing on physical health and AQoL-8D being developed to further enrich psychosocial health).

Recent measures have broadened quality-of-life assessment beyond health, including the ICEpop CAPability measures (ICECAP) with domains such as attachment, stability, achievement, enjoyment and autonomy [17] and the EuroQol Health and Wellbeing instrument (EQ-HWB) [18, 19], which was developed with patients, social care users and unpaid/family carers [19]. The EQ‑HWB and its shorter 9-item version (EQ‑HWB‑9) are currently available as ‘Experimental Version’ instruments to approved research collaborators and do not include an Easy English/Read version [20]. This may limit their suitability for people with disability, where accessible formats support inclusive participation and self‑reporting among respondents with cognitive or communication support needs or lower literacy, thereby reducing avoidable non‑response and exclusion from data collection [21].

The Adult Social Care Outcomes Toolkit (ASCOT) is another preference-based measure developed to assess Social Care–Related Quality of Life (SCRQoL) [22], and is well-suited to settings involving ongoing functional support needs, including for older people and people with disability. ASCOT-SCT4 is the self-complete version, focusing on assessing independence, the adequacy of support in meeting essential daily needs, and the extent to which care is delivered in a manner that preserves personal dignity [22], whereas ASCOT-ER was developed for self-report by adults with intellectual disabilities [23].

Despite these advances in broader, beyond‑health outcome measurement, there remains a need for instruments designed specifically to reflect the experiences and priorities of people with disability. These gaps have important implications for disability policy, where economic evaluation increasingly informs funding and resource allocation. Australia provides a salient example. In 2013, the Australian Government introduced the National Disability Insurance Scheme (NDIS) [24]. NDIS provides eligible individuals aged under 65 with permanent and significant disability with an individualised budget to support their needs [24, 25]. By 2025, the NDIS supported more than 730,000 people with permanent and significant disability, around 3% of the Australian population [26]. Although the NDIS Outcomes Framework reports outcomes across multiple domains, it has a limited scope for measuring and monitoring wellbeing and does not provide preference-weighted outcomes or a psychometrically valid summary score suitable for priority setting [27].

To address these limitations, the Disability Wellbeing Index (DWI) was developed as a preference-based measure informed by consultations with adults under 65 and young people (15–24 years) with disability, qualitative research, and empirical analysis of participant outcomes and survey data [27]. Although developed within the Australian policy context, the DWI was designed to capture broader dimensions of wellbeing that extend beyond health and social care service use, enabling quantification of changes associated with access to supports or policy changes across diverse life domains [27]. Consistent with a social model of disability, wellbeing outcomes are conceptualised as reflecting not only the provision of individual support but also the accessibility and inclusiveness of the surrounding environment.

While ASCOT-SCT4/ER and the DWI both assess quality of life and wellbeing beyond health, they differ in scope and underlying conceptual frameworks. ASCOT-SCT4/ER was developed to measure SCRQoL domains identified as important by people using social care services in the UK, including control over daily life, food and drink, and dignity in care-related interactions [22]. In contrast, the DWI was developed as a disability-specific wellbeing measure reflecting priorities identified by people with disability in Australia [27]. A further distinction concerns the target population of the measures. ASCOT-SCT4/ER was not designed for individuals under 18 years of age [22]. In contrast, the DWI was developed with adults and young people with disability, including those aged 15–24 years [27].

Although both instruments generate utility scores and use a recall period focused on the respondent’s current situation, they differ in scale interpretation and valuation frameworks. In ASCOT-SCT4/ER, 1.00 indicates an ideal state across all domains, while 0.00 is equivalent to death and negative values (− 0.01 to − 0.17) [19] represent states worse than death [22]. In contrast, the DWI employs a 0–1 scale, where 0 is the worst well-being state, and 1 is the best [27]. Elicitation methods and source populations also differ: the DWI utilised Discrete Choice Experiments (DCE) to capture preferences directly from people with disability in Australia [22]. In contrast, the ASCOT-SCT4/ER relied on Best-Worst Scaling (BWS) and Time Trade-Off (TTO) methods to elicit preferences from the UK general population and older adults using services, reporting no substantive differences; the weights were derived from the general population [22].

This study aims to assess the psychometric properties of the DWI and compare its performance with the established preference-based SCRQoL measure, ASCOT-SCT4/ER, among people with disability participating in the Australian NDIS. More specifically, this study examined construct validity through score- and item-level associations, exploratory latent structure, and hypothesis-based known-groups comparisons. This study represents a complementary stage in the evaluation of the DWI, extending previous development and validation work [27] by examining its relationship with ASCOT-SCT4/ER and assessing construct validity using multiple psychometric approaches. In doing so, this paper ensures that outcomes valued by people with disability are appropriately measured and incorporated into research, policy, and resource allocation. Understanding the strengths and limitations of different instruments is particularly important when findings inform funding prioritisation and policy decisions.

## Methods

### Study design and participants

A cross-sectional, screen reader–friendly survey was conducted between July and September 2024 using Qualtrics and distributed by the NDIA via email to more than 40,000 NDIS participants (aged  ≥  15 years) or their nominated supporters. To enhance accessibility, participants could also complete the survey by telephone, Zoom interview, or mailed hard copy. Where participants could not respond, proxies answered as they believed the participant would respond. Ethics approval was granted by the Monash University Human Research Ethics Committee (Project ID: 42195).

### Instruments

The survey comprised five sections, including the DWI, ASCOT-SCT4/ER, the NDIS Short Form Outcomes Framework (SFOF) questionnaire [28], and demographic questions. The DWI was offered in Standard and Easy English (EE) versions. Participants completed either the ASCOT-SCT4 or ASCOT-ER, depending on their communication needs and preferences. Demographic data included age, gender, language spoken at home, marital status, employment, state of residence, Indigenous status, and disability group; for proxy respondents, gender and relationship to the participant were also recorded.

The DWI-Standard and DWI-EE comprise 14 items across 10 domains (relationships, daily life, meaningful life, housing, health, learning, respect and dignity, safety, finances, and work). It also includes a ‘Not Applicable’ option for three relationship items (family, friends, support); when selected, a follow-up question is used to derive responses for scoring [29]. With 14 items, each having five response levels, the DWI defines 5^14^ (≈ 6 billion) possible wellbeing states [22].

ASCOT-SCT4 and ASCOT-ER cover eight domains (control over daily life, personal cleanliness and comfort, food and drink, personal safety, social participation and involvement, occupation, accommodation cleanliness and comfort, and dignity). It uses nine questions with four response levels, defining 4^8^ (≈ 65,000) quality-of-life states [22]. For Dignity in ASCOT-SCT4 and Safety in ASCOT-ER domains, each of which includes two items, only one item response is used in deriving the preference-weighted score. DWI-Standard and DWI-EE versions are available for download free of charge from the DWI website (https://dwi.org.au). ASCOT-SCT4 and ASCOT-ER are available from the ASCOT website (https://research.kent.ac.uk/ascot). Use of ASCOT instruments requires a licence; however, licences for not-for-profit research are available free of charge upon application.

### Psychometric analyses

(i) Convergent and divergent validity

Multiple hypotheses were developed regarding the relationship between the instruments, informed by the alignment of items and dimensions between instruments as outlined.

#### Hypothesis 1a

The item content of the instruments overlaps, and therefore, their overall scores will be strongly correlated (0.60 ≤ ρ ≤ 0.79) [30].

#### Hypothesis 1b

The correlation between DWI and ASCOT-SCT4/ER scores is expected to be stronger among adults, as ASCOT-SCT4/ER was originally developed and primarily validated within adult social care populations, particularly those receiving long-term support [22].

#### Hypothesis 2

The relationship between DWI and ASCOT-SCT4/ER scores is attributable to the extent to which their respective item content overlaps. Specifically, we expected a strong positive correlation (0.60 ≤ ρ ≤ 0.79) [30] for the following conceptually aligned item–domain pairs:

- *DWI Safety with ASCOT-SCT4/ER Safety.*
- *DWI Personal Care with ASCOT-SCT4/ER Personal cleanliness and comfort.*
- *DWI Relationships–Friends with ASCOT-SCT4/ER Social participation and involvement.*
- *DWI Work with ASCOT-SCT4/ER Occupation.*
- *DWI Meaningful Life with ASCOT-SCT4/ER Occupation.*
- *DWI Relationships-Support Team with ASCOT-SCT4/ER Dignity.*

Moderate positive correlation (0.4 ≤ ρ ≤ 0.59) [30] is expected for partially aligned item–domain pairs, including:

- *DWI Housing with ASCOT-SCT4/ER Accommodation cleanliness and comfort.*
- *DWI Dignity with ASCOT-SCT4/ER Dignity.*

Weak to very weak correlations (ρ ≤ 0.39) [30] are expected for item–domain pairs with limited or no conceptual correspondence, including:

- *ASCOT-SCT4/ER Food and drink with all DWI domains.*
- *DWI domains: Finances, Physical Health, and Mental Health with all ASCOT-SCT4/ER items.*

To assess the extent of correlations between the instruments, we calculated Spearman’s (rank-order) correlations both between overall ASCOT-SCT4/ER and DWI scores and between their items. Spearman correlations were selected due to the ordinal nature of item responses and potential non-normal distribution of scores. We interpreted correlation strength using Evans’ guidelines: values of 0.00–0.19 were considered very weak, 0.20–0.39 weak, 0.40–0.59 moderate, 0.60–0.79 strong, and 0.80–1.00 very strong [30]. We assessed correlations separately for ASCOT-SCT4 with DWI-Standard and ASCOT-ER with DWI-EE, as well as jointly and separately for young (15–24 years) and adult (≥ 25 years) cohorts. The 25-year age cut-off aligns with the NDIS Outcomes Framework, which groups ages to reflect differing priorities and outcome domains across life stages [28].

(ii) Structural validity and conceptual overlap

To explore latent structure and conceptual overlap between the Disability Wellbeing Index (DWI) and ASCOT‑SCT4/ER, we conducted an exploratory factor analysis (EFA) [31] in EViews (Version 14). Factor retention was guided by multiple criteria, including the minimum average partial (MAP) test, parallel analysis, scree plot inspection, model fit indices (Schwarz (BIC), RIF, NNFI (TLI) and CFI), and substantive interpretability. These criteria were considered jointly rather than hierarchically. The final solution was selected based on improved model fit, meaningful factor loadings, and conceptual coherence with the hypothesised distinction between broader disability‑related wellbeing and SCRQoL. As latent constructs were expected to correlate, an oblique rotation using the Promax method was applied. Factor loadings of ≥ 0.30 were considered meaningful [32].

(iii) Known-groups validity

The DWI and ASCOT-SCT4/ER will differ, in the expected direction, between groups whose wellbeing is anticipated to be different. Specifically, based on the availability of four relevant items from the SFOF, we hypothesised that both instruments would show higher scores among participants reporting better self-rated health [33], undertaking meaningful leisure activities [34, 35], greater self-advocacy [36], and increased decision-making autonomy [37].

Self-rated health was grouped into four categories (poor/fair, good, very good, excellent), with poor/fair used as the reference group due to small cell sample sizes. Decision-making autonomy was grouped into four categories (none/a little of the time, some of the time, most of the time, all of the time), with none/a little of the time used as the reference group. Meaningful leisure activities and self-advocacy were classified as binary variables (no/yes), with no as the reference category. This regression-based known-groups approach is consistent with previous validation studies of preference-based measures, which assess whether scores differ across predefined groups expected to vary in health or wellbeing while controlling for confounders [38, 39].

To enable cross-instrument comparisons, both outcome measures were standardised [40] to have a mean of 0 and a standard deviation of 1, allowing effects to be interpreted as differences in conditional means in standard-deviation units. Separate linear regression models were estimated for each outcome, with group indicators as predictors and adjustment for age, gender, disability type, and proxy respondent status. Evidence of known-groups validity was inferred if scores differed across groups in the expected direction (higher scores among respondents reporting better health, greater autonomy, meaningful leisure, and self-advocacy). We also ran analyses separately for the ASCOT-SCT4 with DWI-Standard comparison and the ASCOT-ER with DWI-EE comparison. All analyses were conducted in Stata 18 (StataCorp LLC, College Station, TX).

## Results

### Sample characteristics

The survey link was accessed 3,986 times; 479 did not proceed to the explanatory statement. After excluding 81 respondents who did not provide explicit consent, 3,426 responses remained. Of these, 1,221 did not complete all 14 DWI items and were excluded because a DWI score could not be calculated. A further 334 respondents who did not complete all ASCOT-SCT4/ER items were excluded, leaving 1871 participants (Appendix 1).

The mean age was 40 years (SD = 18). Most participants spoke English only (93%), and 4% identified as First Nations Australians (Aboriginal and/or Torres Strait Islander). Participants identified as male (44%), female (50%), or non-binary (4%). Disability groups were well represented, particularly psychosocial (46%), autism (42%), intellectual (38%), physical (36%), and sensory (33%) (Table 1). We reported additional sample characteristics in Appendix 2, and mean and median DWI and ASCOT-SCT4/ER utility scores by disability group are presented in Appendix 3. The comparison of demographic and disability characteristics between the excluded sample and the study sample is also given in Appendix 7.

**Table 1:** Sample characteristics

	Freq/mean	Prop/(SD)
Age, Mean (SD) (*N* = 1854)	40.2	(18.4)
First Nations (%) (*N* = 1860)	79	4%
Speaks English only (%) (*N* = 1838)	1702	93%
Gender (*N* = 1871) (%)		
Male	828	44%
Female	942	50%
Non-binary	72	4%
Prefer not to say	29	2%
Disability groups* (*N* = 1871)		
Sensory	615	33%
Intellectual	745	40%
Physical	761	41%
Psychosocial	888	47%
Head Injury, Stroke, Acquired Brain Injury	272	15%
Autism	787	42%
Other	140	8%

*Percentages add up to more than 100% because participants could tick more than one option.

### Convergent and divergent validity

#### Overall score correlations between DWI and ASCOT-SCT4

**Fig. 1:**
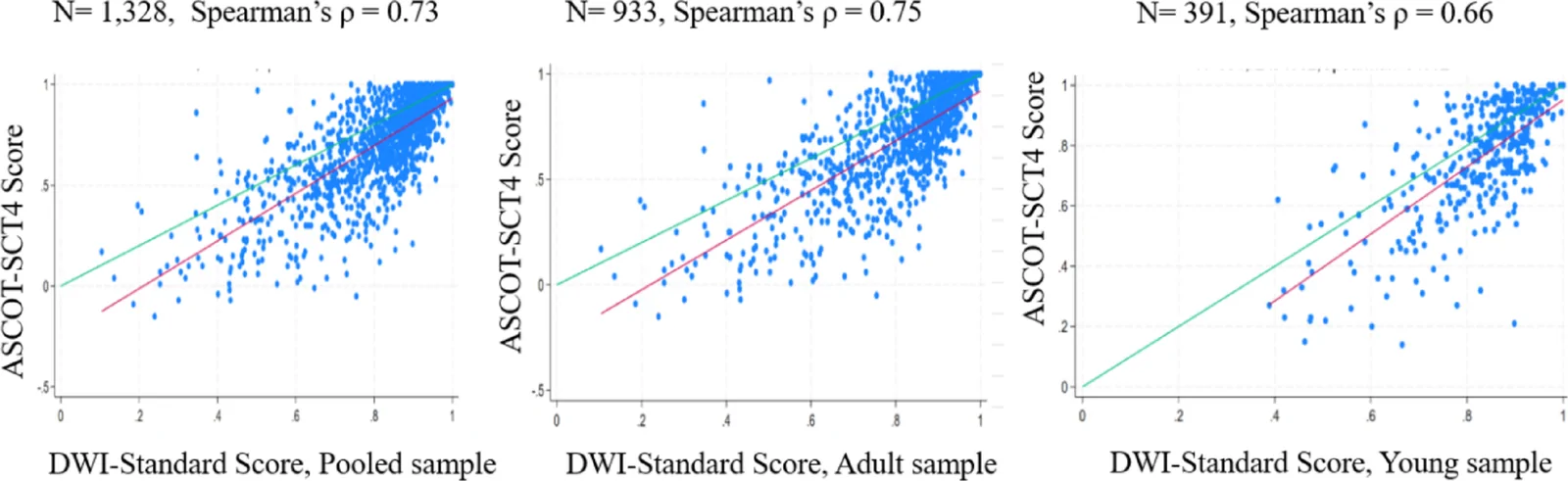
Correlations between DWI-Standard and ASCOT-SCT4 scores The red line denotes the fitted linear regression line, whereas the green line represents the theoretical line of perfect agreement

**Fig. 2:**
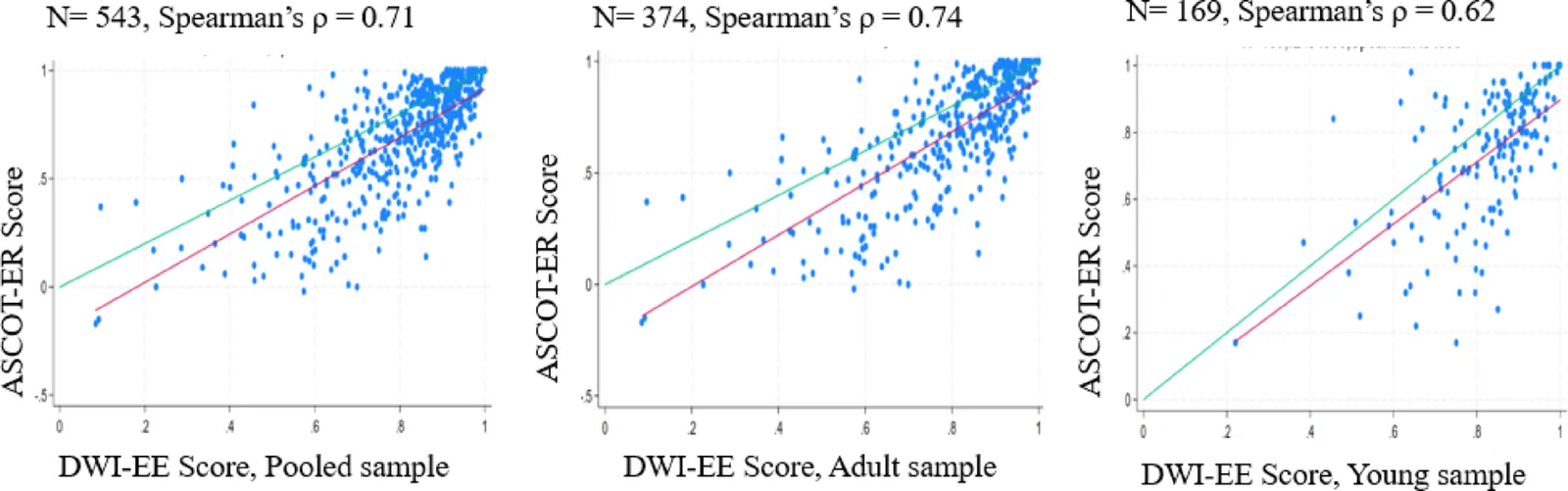
Correlations between DWI-EE and ASCOT-ER scores The red line denotes the fitted linear regression line, whereas the green line represents the theoretical line of perfect agreement

Consistent with Hypothesis 1a, utility scores were strongly correlated for both ASCOT-SCT4 with DWI-Standard (ρ = 0.73, *p* < 0.001) and ASCOT-ER with DWI-EE (ρ = 0.71, *p* < 0.001). Figures 1 and 2 present scatter plots for the pooled sample and by age cohorts. Consistent with Hypothesis 1b, correlations between instrument scores were slightly lower for younger participants than for adults for both between DWI-Standard and ASCOT-SCT4 (0.66 vs. 0.75) and between DWI-EE and ASCOT-ER (0.62 vs. 0.74). Nevertheless, all correlations exceeded 0.60, indicating strong associations across both cohorts.

#### Item response correlations between DWI and ASCOT-SCT4/ER

For both DWI-Standard with ASCOT-SCT4 and DWI-EE with ASCOT-ER, most item-level correlations were moderate or weak. For DWI-Standard and ASCOT-SCT4, the strongest correlations were observed for Safety (ρ = 0.58), Occupation/Meaningful life (ρ = 0.56), Personal cleanliness and comfort/Personal care (ρ = 0.53), and Social participation and involvement/Relationships—Friends (ρ = 0.53). These findings support the hypothesised pattern of convergent validity based on conceptual overlap, although none reached the threshold for strong correlations specified in Hypothesis 2 (Table 2).

Consistent with Hypothesis 2, ASCOT-SCT4 Accommodation cleanliness and comfort demonstrated modest correlations with the DWI Housing domain (ρ = 0.47), reflecting that housing wellbeing encompasses more than cleanliness and comfort (for example, who you live with). As expected, DWI domains without direct ASCOT-SCT4 counterparts, such as Finances, Physical Health and Mental Health, showed weak correlations with ASCOT-SCT4 items, supporting divergent validity. However, contrary to Hypothesis 2, the ASCOT-SCT4 Dignity item showed only weak correlations with the DWI Respect and Dignity (ρ = 0.27) and Relationship-Support Team (ρ = 0.33) items.

For DWI-EE and ASCOT-ER, the highest correlations were observed for Safety (ρ = 0.55), Social Participation and Involvement/Relationship–Friends (ρ = 0.52), and Occupation/Meaningful Life (ρ = 0.51), followed by Personal Cleanliness and Comfort/Personal Care (ρ = 0.50), and Social Participation and Involvement/Meaningful Life (ρ = 0.50) (Table 2). While consistent with the hypothesised pattern of convergent validity, none met the threshold for strong correlations specified in Hypothesis 2. Contrary to expectations, correlations between Occupation/Work (ρ = 0.39), and between Accommodation Cleanliness and Comfort/ Housing (ρ = 0.37) were weaker than anticipated (Table 2). See Appendix 4 for further correlation coefficients by age cohorts.

**Table 2:** Spearman correlations between DWI and ASCOT-SCT4/ER items

		Relationship			Personal care	Everyday activities	Meaningful life	Housing	Physical health	Mental health	Learning	Respect & Dignity	Safety	Finances	Work
		Family	Friends	Support team											
		DWI-Standard version (*N* = 1226, age pooled sample)													
ASCOT -SCT4	Control over daily life	0.21	0.29	0.25	0.30	0.31	0.38	0.33	0.23	0.30	0.34	0.30	0.35	0.29	0.33
	Personal cleanliness and comfort	0.28	0.32	0.27	0.53**↓**	0.41	0.38	0.39	0.32	0.38	0.30	0.30	0.37	0.31	0.30
	Food and drink	0.34	0.25	0.22	0.37	0.33	0.32	0.38	0.26	0.27	0.23	0.30	0.33	0.35	0.25
	Safety	0.37	0.30	0.26	0.30	0.34	0.35	0.41	0.34	0.37	0.24	0.37	0.58**↓**	0.39	0.32
	Social participation and involvement	0.30	0.53**↓**	0.29	0.33	0.43	0.47**↓**	0.37	0.33	0.41**↑**	0.38	0.37	0.39	0.36	0.38
	Occupation	0.31	0.37	0.27	0.35	0.45	0.56**↓**	0.43	0.37	0.41**↑**	0.42	0.36	0.41	0.40**↑**	0.44**↓**
	Accommodation cleanliness, and comfort	0.38	0.29	0.29	0.38	0.42	0.38	0.47	0.35	0.35	0.25	0.34	0.41	0.40**↑**	0.28
	Dignity	0.20	0.17	0.33**↓**	0.15	0.24	0.27	0.24	0.22	0.22	0.18	0.27↓	0.28	0.17	0.19
		DWI-EE version (*N* = 500, age pooled sample)													
ASCOT -ER	Control over daily life	0.38	0.33	0.35	0.31	0.39	0.43	0.39	0.32	0.38	0.37	0.38	0.36	0.34	0.35
	Personal cleanliness and comfort	0.42	0.36	0.34	0.50**↓**	0.44	0.37	0.40	0.39	0.42	0.31	0.37	0.39	0.36	0.32
	Food and drink	0.32	0.22	0.26	0.30	0.27	0.29	0.34	0.26	0.3	0.21	0.33	0.41	0.35	0.29
	Accommodation cleanliness, and comfort	0.36	0.23	0.28	0.34	0.37	0.29	0.37**↓**	0.30	0.33	0.25	0.30	0.38	0.38	0.32
	Safety	0.39	0.41	0.30	0.34	0.40	0.32	0.42	0.33	0.44	0.23	0.41	0.55**↓**	0.39	0.31
	Social participation and involvement	0.43	0.52**↓**	0.38	0.38	0.45	0.50**↓**	0.40	0.37	0.41	0.43	0.45	0.40	0.30	0.42
	Occupation	0.37	0.38	0.31	0.37	0.44	0.51**↓**	0.41	0.38	0.40	0.41	0.38	0.41	0.34	0.39**↓**
	Dignity	0.29	0.26	0.44**↓**	0.21	0.23	0.25	0.32	0.17	0.27	0.23	0.34**↓**	0.25	0.16	0.20

↓ indicates correlations that were weaker than hypothesised. ↑ indicates correlations that were stronger than hypothesised

### Structural validity and conceptual overlap

The MAP test suggested a two-factor solution for DWI-Standard and ASCOT-SCT4. The two-factor solution showed clear advantages across several fit criteria over the user-specified one-factor solution (ΔCFI = + 0.03; lower AIC and BIC values). These statistical improvements were further supported by the parallel analysis and the scree plot (Appendix 5, Fig. A5.1), both indicating that two factors should be retained. Conceptually, the two-factor structure was also the most interpretable, comprising a general wellbeing factor and a SCRQoL factor. On this basis, the two-factor model was selected as the most parsimonious and theoretically coherent representation of the data.

Factor loadings indicated both conceptual differentiation alongside overlap between the instruments. DWI items generally loaded more strongly on the general wellbeing factor, whereas ASCOT-SCT4 items loaded more strongly on the SCRQoL factor. Several items from both instruments also showed cross-loadings on the alternative factor, indicating shared variance across domains (See Table A5.1, Appendix 5).

Similarly, for DWI-EE and ASCOT-ER, the MAP test, parallel analysis and scree plot (Appendix 5, Fig. A5.2) suggested a two-factor structure. DWI-EE items generally loaded predominantly on a general wellbeing factor, although Safety and Finances showed notable cross-loadings on the SCRQoL factor. ASCOT-ER items loaded primarily on the SCRQoL factor, with Social participation and involvement showing a stronger loading on the general wellbeing factor. Overall, the pattern of loadings suggests related but distinct constructs with some overlap rather than a fully separable structure (See Table A5.2, Appendix 5).

### Known-groups validity

Both instruments differentiated between the pre-specified known groups, supporting their construct validity consistent with Hypothesis 3 (Table 3). Based on the magnitude of the estimated standardised coefficients, ASCOT-SCT4 exhibited larger differences across sub-groups by participating in meaningful leisure activities, decision-making autonomy, and self-advocacy, whereas the DWI showed a greater discriminatory power for self-rated health. For example, a coefficient of 0.58 for ‘good’ self-rated health in the DWI Standard model indicates that, on average, these respondents scored 0.58 SD higher than those reporting poor or fair health. To aid interpretation of these adjusted regression results, unadjusted mean ± SD for ASCOT-SCT4, DWI-Standard, ASCOT-ER, and DWI-EE across each SFOF comparator group are presented in Appendix Table A6.1.

**Table 3:** Known-groups validity: regression analysis of standardised ASCOT-SCT4/ER and DWI scores across comparator groups

Explanatory variables\standardised outcomes		ASCOT-SCT4 Score	DWI-Standard score	ASCOT-ER Score	DWI-EE Score
N		1312		536	
Self-rated health (Ref: Poor/Fair)	Good	0.406*** (0.060)	0.583*** (0.060)	0.310*** (0.087)	0.472*** (0.097)
	Very Good	0.621*** (0.063)	0.875*** (0.063)	0.589*** (0.096)	0.799*** (0.100)
	Excellent	0.905*** (0.070)	1.152*** (0.071)	0.709*** (0.112)	0.925*** (0.116)
Meaningful Leisure activities (Ref: No)	Yes	0.676*** (0.052)	0.555*** (0.052)	0.582*** (0.077)	0.568*** (0.080)
Decision-making autonomy (Ref: None/a little of the time)	Some of the time	0.241*** (0.060)	0.193*** (0.060)	0.171* (0.093)	0.282*** (0.097)
	Most of the time	0.490*** (0.057)	0.381*** (0.057)	0.532*** (0.090)	0.435*** (0.094)
	All of the time	0.691*** (0.065)	0.452*** (0.065)	0.924*** (0.104)	0.577*** (0.108)
Self-advocacy (Ref: No)	Yes	0.260*** (0.044)	0.198*** (0.044)	0.223*** (0.070)	0.199*** (0.073)

*** *p* < 0.01, ** *p* < 0.05, * *p* < 0.10. Controlled by age, gender, disability type, and proxy status. ASCOT and DWI scores are standardised to have a mean of 0 and a standard deviation of 1.

## Discussion

This study examined the psychometric properties of the DWI by comparing it with the ASCOT-SCT4/ER using data from NDIS participants aged 15 years and over. Findings indicate that these two instruments show conceptual overlap, yet they are not substitutes.

DWI and ASCOT-SCT4/ER scores were strongly and positively correlated across age cohorts and survey formats, indicating substantial conceptual overlap between the constructs captured by the instruments. Correlations were higher among adults than younger participants. This may partly reflect differences in the target populations and development contexts of the instruments: ASCOT-SCT4 and ASCOT-ER are adult SCRQoL measures, with evidence of use among adults with care and support needs [22, 23, 41], whereas the DWI was developed with adults and young people with disability, including those aged 15–24 years [27]. It may also reflect life-stage differences in the salience of wellbeing domains, as recent disability-specific evidence suggests that the relative importance of life domains differs between young people and adults with disability [11].

Item-level correlations were moderate between aligned domains. Correlations were generally stronger among adults for ASCOT-SCT4 with DWI-Standard compared to ASCOT-ER with DWI-EE. Despite similar labels, dignity-related items correlated much more weakly than expected. The DWI dignity item reflects being treated and spoken to with respect across social contexts, whereas the ASCOT-SCT4/ER dignity item focuses on interactions with paid support staff. Although the ASCOT-SCT4/ER dignity item correlated more strongly with the DWI Relationships–Support team item than with the DWI dignity item, the association remained weak. This suggests that similarly labelled domains may operationalise distinct constructs, with implications for interpretation across instruments, subgroups, and survey contexts.

Exploratory factor analysis further suggested that DWI and ASCOT-SCT4/ER measure related but non-identical constructs, with DWI items generally loading more strongly on a broader wellbeing factor and ASCOT-SCT4/ER items tending to load more strongly on a SCRQoL factor. The presence of cross-loadings across several items indicates areas of shared variance between the instruments, particularly in domains such as social participation, safety, and living conditions. These exploratory findings suggest conceptual overlap and some differentiation between the instruments, but should be interpreted cautiously because the combined-item EFA was intended to examine overlap between instruments rather than establish the standalone structural validity of the DWI. Initial DWI-only structural analyses are reported elsewhere [24].

Known-groups analyses also supported construct validity, with both instruments distinguishing between groups expected to differ in wellbeing. DWI scores showed larger differences by self-rated health than SCT4/ER, likely reflecting the unique health-related items included in DWI but not in SCT4/ER. This finding should be interpreted as evidence that health is embedded within the DWI’s broader disability-wellbeing construct, rather than as evidence that the DWI functions as a conventional HRQoL measure. In the present study, ASCOT-SCT4/ER showed larger differences by meaningful leisure, decision-making autonomy and self-advocacy, which is consistent with its more specific focus on SCRQoL [22].

Overall, the study showed that DWI has meaningful convergence with ASCOT-SCT4/ER while also suggesting that the instruments emphasise somewhat different aspects of quality of life and wellbeing. This pattern is consistent with previous ASCOT-SCT4 validation studies showing stronger associations between ASCOT-SCT4 and broader wellbeing or capability measures than between ASCOT-SCT4 and HRQoL measures. Rand et al. [41] reported that ASCOT-SCT4 was more strongly correlated with ICECAP-A/O, ρ = 0.62 and ρ = 0.67, than with EQ-5D-3L, ρ = 0.37, supporting the view that social care-related quality of life overlaps more closely with broader capability and wellbeing constructs than with HRQoL alone. Similarly, in a study of frail older adults in the Netherlands [42], the Dutch translation of ASCOT-SCT4 showed a stronger correlation with ICECAP-O, ρ = 0.63, than with EQ-5D-3L, ρ = 0.41. Taken together, these findings suggest that the DWI may be more closely aligned with broader wellbeing and SCRQoL constructs than with narrower HRQoL constructs such as those captured by EQ-5D. This interpretation is consistent with recent Australian adolescent evidence showing weak to moderate correlations and limited latent overlap between DWI and HRQoL measures, including CHU9D and EQ-5D-5L, suggesting that HRQoL and subjective wellbeing measures provide complementary rather than substitutable information [43]. However, as the present study did not include an HRQoL measure, future studies comparing DWI directly with HRQoL measures are needed to clarify these relationships.

These findings suggest that instrument choice should be guided by research and policy aims. DWI and ASCOT-SCT4/ER scores were strongly correlated, indicating related constructs, but item-level and known-groups findings suggest some differences in the aspects of quality of life and wellbeing emphasised by each measure. This matters for economic evaluation because alternative preference-based instruments can produce different cost-effectiveness conclusions [44, 45]. In this context, the DWI provides a disability-specific preference-based measure that captures health alongside broader life-domain outcomes relevant to disability supports and policy, whereas ASCOT-SCT4/ER offers a well-established measure of SCRQoL, grounded in the capability approach and widely applied internationally to evaluate the outcomes of social care services and interventions [46].

Furthermore, in applied evaluations, both measures can be analysed at the overall-score level and at the domain/item level to identify which aspects of quality of life/wellbeing are driving observed differences. For ASCOT-SCT4/ER, this approach has been used in prior studies [47–49]. However, domain/item profiles should be interpreted as complementary to the full preference-weighted index rather than as substitutes for it. Analysing only one or a small number of items may reduce sensitivity because individual items have restricted response ranges and may show limited variation compared with the full index [46]. The DWI may nevertheless provide useful descriptive information in evaluations of interventions outside formal care. For example, in education- or skills-focused interventions, the Learning domain may capture important effects even when changes in overall wellbeing are modest.

Several limitations of the study should be noted. First, the sample may not be fully representative of all people with disability in Australia, as it was limited to NDIS participants and excludes a substantial proportion of people with disability not receiving NDIS support. It may also underrepresent those with lower literacy or limited access to, or engagement with, email-based NDIA communications, limiting generalisability. Second, the cross-sectional design precludes assessment of sensitivity to change over time. Future research could examine longitudinal use of the DWI to assess individual- and population-level change across broader domains relative to ASCOT-SCT4/ER. As ASCOT-SCT4/ER was developed for adults, comparisons involving participants aged 15–17 years should be interpreted with caution. Third, as we compared only the DWI and ASCOT-SCT4/ER, we cannot assess their relative performance against other preference-based measures; studies including other measures (e.g., EQ-HWB, EQ-5D, ICECAP) would help clarify relative strengths and suitability for different decision contexts. A further limitation is that, although people with lived experience, family members, carers, and community representatives contributed to the broader development of the DWI through consultations, qualitative interviews, and preference elicitation studies that informed item selection and valuation, they were not directly involved in the design, analysis, interpretation, or reporting of the present study.

## Conclusion

These findings provide initial evidence supporting the construct validity of the DWI and highlight its conceptual differentiation from the ASCOT-SCT4/ER. Future research should extend this work using additional comparator measures and longitudinal designs. Developed with people with disability, the DWI provides a preference-based wellbeing outcome that captures health alongside broader life-domain outcomes relevant to disability supports and policy, whereas ASCOT-SCT4/ER offers a well-established measure of SCRQoL, grounded in the capability approach and widely applied internationally to evaluate the outcomes of social care services and interventions. The choice of instrument should be guided by research and policy aims.

## Supplementary Information

Below is the link to the electronic supplementary material.

Supplementary Material 1

## Data Availability

The data generated and analysed during the current study are not publicly available due to ethical and data governance restrictions but may be available from the corresponding author on reasonable request, subject to appropriate approvals.
