# Supplementary material for: Psychometric evaluation of the Disability Wellbeing Index (DWI) against the Adult Social Care Outcomes Toolkit (ASCOT) among people with disability
Source: Qual Life Res. 2026 Sep 27;35(10):301. doi: 10.1007/s11136-026-04406-6 (PMC13616849; doi:10.1007/s11136-026-04406-6)
Supplement: Supplementary file 1 [file 11136_2026_4406_MOESM1_ESM.docx]

*Quality of Life Research*

**Psychometric Evaluation of Disability Wellbeing Index (DWI) and Adult Social Care Outcomes Toolkit (ASCOT) Among People with Disability**

Online Supplementary Material

Gozde Aydin^1*^, Samia Badji^1^, Dennis Petrie^1^, Anthony Harris^1^, Gang Chen^1,2^

1 Centre for Health Economics, Monash University, Victoria, Australia

2 Centre for Health Policy, The University of Melbourne, Victoria, Australia

*Corresponding author: gozde.aydin@monash.edu

## **Appendix 1. Flow Chart – Sample selection**

**Figure A1.** Flow chart - sample selection

## **Appendix 2. Detailed Sample Characteristics**

**Table A2.** Sample characteristics

|  | **Freq/mean** | **Prop/(SD)** |
| --- | --- | --- |
| **Age,** Mean (SD) (N=1854) | 40.2 | 18.4 |
| **First Nations** (%) (N=1860) | 79 | 4% |
| **Speaks English only** (%) (N=1838) | 1702 | 93% |
| **Gender** (N=1871) (%) |  |  |
| Male | 828 | 44% |
| Female | 942 | 50% |
| Non-binary | 72 | 4% |
| Prefer not to say | 29 | 2% |
| **Marital status** (N=1861) |  |  |
| Married or de facto, or having a partner | 450 | 24% |
| Single | 1101 | 59% |
| Widowed/divorced/separated | 248 | 13% |
| Prefer not to say | 62 | 3% |
| **Education** (N=1859) |  |  |
| No school / Year 8 or less | 62 | 3% |
| High school | 765 | 41% |
| Diploma or certificate or similar | 307 | 17% |
| University and over | 330 | 18% |
| Other | 349 | 19% |
| Prefer not to say | 46 | 3% |
| **Employment*** (N=1855) |  |  |
| Full-time | 110 | 6% |
| Part-time | 247 | 13% |
| Unemployed | 446 | 24% |
| Retired/pensioner | 533 | 29% |
| Home/duties/home carer | 96 | 5% |
| Student | 332 | 18% |
| Other | 342 | 18% |
| **Disability groups*** (N=1871) |  |  |
| Sensory | 615 | 33% |
| Intellectual | 745 | 40% |
| Physical | 761 | 41% |
| Psychosocial | 888 | 47% |
| Head Injury, Stroke, Acquired Brain Injury | 272 | 15% |
| Autism | 787 | 42% |
| Other | 140 | 8% |
| **Response type (**N=1848) | **DWI-EE (N=536)** | **DWI-Std (N=1312**) |
|  | N (%) | |
| NDIS participant without support | 225 (42) | 607 (46) |
| NDIS participant with support | 78 (15) | 83 (6) |
| Proxy helping NDIS participant | 38 (7) | 64 (5) |
| Proxy on behalf of the NDIS participant | 195 (36) | 558 (42) |

* Percentages add up to more than 100% because participants could tick more than one option.

## **Appendix 3. Comparison of mean +/- SD and median utilities according to disability groups**

**Table A3.** Comparison of mean +/- SD and median (IQR) utilities according to disability groups

|  |  | **DWI-Standard** | | **ASCOT-SCT4** | |  | **DWI-EE** | | **ASCOT-ER** | |
| --- | --- | --- | --- | --- | --- | --- | --- | --- | --- | --- |
| **Disability group** | **N** | **Mean ± SD** | **Median** | **Mean ± SD** | **Median** | **N** | **Mean ± SD** | **Median** | **Mean ± SD** | **Median** |
| Sensory | 395 | 0.791 ± 0.160 | 0.842 | 0.680 ± 0.244 | 0.730 | 214 | 0.784 ± 0.165 | 0.837 | 0.621 ± 0.237 | 0.690 |
| Intellectual | 447 | 0.835 ± 0.133 | 0.875 | 0.746 ± 0.216 | 0.800 | 259 | 0.795 ± 0.167 | 0.846 | 0.635 ± 0.242 | 0.690 |
| Physical | 489 | 0.789 ± 0.156 | 0.836 | 0.653 ± 0.248 | 0.700 | 188 | 0.780 ± 0.166 | 0.824 | 0.604 ± 0.266 | 0.665 |
| Psychosocial | 600 | 0.754 ± 0.163 | 0.796 | 0.639 ± 0.250 | 0.685 | 256 | 0.747 ± 0.175 | 0.789 | 0.582 ± 0.248 | 0.625 |
| ABI | 174 | 0.818± 0.147 | 0.864 | 0.726 ± 0.241 | 0.780 | 95 | 0.804 ± 0.161 | 0.840 | 0.656 ± 0.248 | 0.750 |
| Autism | 569 | 0.784 ± 0.154 | 0.832 | 0.684 ± 0.238 | 0.730 | 217 | 0.783 ± 0.158 | 0.836 | 0.620 ± 0.230 | 0.680 |
| Other | 281 | 0.822 ± 0.141 | 0.866 | 0.712 ± 0.235 | 0.745 | 105 | 0.780 ± 0.181 | 0.855 | 0.602 ± 0.259 | 0.650 |

***Appendix 4. Spearman item response correlations between DWI and ASCOT-SCT4/ER items across age groups***

## **Table A4.1** Spearman item response correlations between DWI-Standard and ASCOT-SCT4 items across adult and young samples

|  |  | **DWI Standard version (Adult sample, N=864)** | | | | | | | | | | | | | | |
| --- | --- | --- | --- | --- | --- | --- | --- | --- | --- | --- | --- | --- | --- | --- | --- | --- |
|  |  | **Relationship** | | | **Personal care** | **Everyday activities** | **Meaningful life** | **Housing** | **Physical health** | **Mental health** | **Learning** | **Respect & Dignity** | **Safety** | **Finances** | **Work** |  |
|  |  | **family** | **friends** | **support team** |  |  |  |  |  |  |  |  |  |  |  |  |
| **ASCOT -SCT4** | **Control over daily life** | 0.22 | 0.31 | 0.28 | 0.33 | 0.34 | 0.42 | 0.35 | 0.23 | 0.33 | 0.36 | 0.31 | 0.37 | 0.31 | 0.33 |  |
|  | **Personal cleanliness and comfort** | 0.30 | 0.33 | 0.32 | 0.53 | 0.42 | 0.39 | 0.39 | 0.31 | 0.39 | 0.30 | 0.31 | 0.37 | 0.29 | 0.30 |  |
|  | **Food and drink** | 0.36 | 0.31 | 0.28 | 0.44 | 0.39 | 0.37 | 0.39 | 0.25 | 0.31 | 0.27 | 0.35 | 0.37 | 0.38 | 0.27 |  |
|  | **Safety** | 0.40 | 0.35 | 0.28 | 0.33 | 0.37 | 0.38 | 0.43 | 0.34 | 0.40 | 0.27 | 0.42 | 0.62 | 0.42 | 0.32 |  |
|  | **Social participation and involvement** | 0.35 | 0.55 | 0.33 | 0.36 | 0.45 | 0.50 | 0.42 | 0.36 | 0.44 | 0.40 | 0.39 | 0.43 | 0.40 | 0.39 |  |
|  | **Occupation** | 0.34 | 0.40 | 0.31 | 0.40 | 0.49 | 0.59 | 0.46 | 0.40 | 0.44 | 0.44 | 0.38 | 0.43 | 0.42 | 0.45 |  |
|  | **Accommodation cleanliness and comfort** | 0.41 | 0.36 | 0.36 | 0.43 | 0.47 | 0.43 | 0.48 | 0.35 | 0.38 | 0.29 | 0.40 | 0.43 | 0.44 | 0.30 |  |
|  | **Dignity** | 0.20 | 0.19 | 0.33 | 0.18 | 0.27 | 0.29 | 0.28 | 0.22 | 0.21 | 0.18 | 0.29 | 0.30 | 0.18 | 0.20 |  |
|  |  | **DWI-Standard version (Young sample, N=362)** | | | | | | | | | | | | | |  |
| **ASCOT -SCT4** | **Control over daily life** | 0.18 | 0.22 | 0.17 | 0.23 | 0.24 | 0.27 | 0.29 | 0.23 | 0.23 | 0.30 | 0.26 | 0.28 | 0.24 | 0.32 |  |
|  | **Personal cleanliness and comfort** | 0.26 | 0.31 | 0.17 | 0.56 | 0.39 | 0.35 | 0.37 | 0.35 | 0.38 | 0.28 | 0.29 | 0.39 | 0.35 | 0.29 |  |
|  | **Food and drink** | 0.31 | 0.16 | 0.13 | 0.23 | 0.23 | 0.14 | 0.32 | 0.25 | 0.19 | 0.12 | 0.22 | 0.20 | 0.30 | 0.21 |  |
|  | **Accommodation cleanliness and comfort** | 0.32 | 0.25 | 0.27 | 0.27 | 0.30 | 0.24 | 0.33 | 0.29 | 0.30 | 0.13 | 0.31 | 0.48 | 0.33 | 0.33 |  |
|  | **Safety** | 0.18 | 0.50 | 0.20 | 0.26 | 0.39 | 0.38 | 0.25 | 0.26 | 0.34 | 0.32 | 0.32 | 0.28 | 0.26 | 0.37 |  |
|  | **Social participation and involvement** | 0.27 | 0.36 | 0.20 | 0.25 | 0.36 | 0.44 | 0.34 | 0.26 | 0.34 | 0.36 | 0.35 | 0.35 | 0.36 | 0.42 |  |
|  | **Occupation** | 0.33 | 0.18 | 0.19 | 0.27 | 0.33 | 0.23 | 0.41 | 0.32 | 0.29 | 0.15 | 0.21 | 0.35 | 0.31 | 0.25 |  |
|  | **Dignity** | 0.22 | 0.14 | 0.35 | 0.09 | 0.18 | 0.20 | 0.13 | 0.18 | 0.23 | 0.17 | 0.21 | 0.21 | 0.15 | 0.16 |  |

**Table A4.2** Spearman item response correlations between DWI-EE and ASCOT-ER items across adult and young samples

|  |  | **DWI-EE (Adult sample, N=342)** | | | | | | | | | | | | | | |
| --- | --- | --- | --- | --- | --- | --- | --- | --- | --- | --- | --- | --- | --- | --- | --- | --- |
|  |  | **Relationship** | | | **Personal care** | **Everyday activities** | **Meaningful life** | **Housing** | **Physical health** | **Mental health** | **Learning** | **Respect & Dignity** | **Safety** | **Finances** | **Work** |  |
|  |  | **family** | **friends** | **support team** |  |  |  |  |  |  |  |  |  |  |  |  |
| **ASCOT -**  **E**  **R** | **Control over daily life** | 0.34 | 0.32 | 0.33 | 0.36 | 0.44 | 0.44 | 0.39 | 0.38 | 0.38 | 0.37 | 0.39 | 0.41 | 0.37 | 0.34 |  |
|  | **Personal cleanliness and comfort** | 0.43 | 0.43 | 0.35 | **0.50** | 0.47 | 0.39 | 0.47 | 0.44 | 0.46 | 0.35 | 0.44 | 0.46 | 0.41 | 0.43 |  |
|  | **Food and drink** | 0.35 | 0.30 | 0.29 | 0.34 | 0.37 | 0.32 | 0.39 | 0.27 | 0.36 | 0.25 | 0.37 | 0.44 | 0.37 | 0.35 |  |
|  | **Safety** | 0.39 | 0.32 | 0.33 | 0.35 | 0.41 | 0.32 | 0.38 | 0.28 | 0.36 | 0.29 | 0.35 | 0.41 | 0.39 | 0.39 |  |
|  | **Social participation and involvement** | 0.46 | 0.47 | 0.31 | 0.36 | 0.44 | 0.36 | 0.48 | 0.37 | 0.45 | 0.27 | 0.42 | 0.61 | 0.42 | 0.34 |  |
|  | **Occupation** | 0.47 | 0.52 | 0.42 | 0.44 | 0.52 | 0.53 | 0.44 | 0.44 | 0.44 | 0.45 | 0.50 | 0.49 | 0.36 | 0.47 |  |
|  | **Accommodation cleanliness and comfort** | 0.38 | 0.39 | 0.35 | 0.42 | 0.52 | 0.56 | 0.43 | 0.42 | 0.44 | 0.41 | 0.40 | 0.44 | 0.37 | 0.38 |  |
|  | **Dignity** | 0.35 | 0.29 | 0.45 | 0.28 | 0.33 | 0.32 | 0.37 | 0.24 | 0.30 | 0.31 | 0.42 | 0.31 | 0.15 | 0.35 |  |
|  |  | **DWI-EE (Young sample, N=158)** | | | | | | | | | | | | | |  |
| **ASCOT -4ER** | **Control over daily life** | 0.48 | 0.35 | 0.38 |  | 0.26 | 0.40 | 0.41 | 0.22 | 0.37 | 0.37 | 0.37 | 0.25 | 0.26 | 0.48 |  |
|  | **Personal cleanliness and comfort** | 0.37 |  | 0.32 | 0.49 | 0.36 | 0.34 | 0.23 | 0.31 | 0.32 |  |  | 0.23 | 0.24 | 0.37 |  |
|  | **Food and drink** | 0.25 |  | 0.21 | 0.21 |  | 0.21 | 0.23 |  |  |  | 0.24 | 0.32 | 0.32 | 0.25 |  |
|  | **Accommodation cleanliness and comfort** | 0.28 |  |  | 0.30 | 0.29 | 0.23 | 0.34 | 0.32 | 0.23 |  |  | 0.29 | 0.36 | 0.28 |  |
|  | **Safety** |  | 0.26 | 0.26 | 0.29 | 0.28 | 0.23 | 0.27 | 0.26 | 0.42 |  | 0.37 | 0.41 | 0.34 | 0.26 |  |
|  | **Social participation and involvement** | 0.31 | 0.51 | 0.26 | 0.21 | 0.25 | 0.41 | 0.31 | 0.23 | 0.31 | 0.40 | 0.30 |  |  | 0.31 |  |
|  | **Occupation** | 0.35 | 0.39 | 0.24 | 0.24 | 0.23 | 0.38 | 0.35 | 0.25 | 0.29 | 0.41 | 0.34 | 0.31 | 0.25 | 0.35 |  |
|  | **Dignity** | 0.48 | 0.35 | 0.38 |  | 0.26 | 0.40 | 0.41 | 0.22 | 0.37 | 0.37 | 0.37 | 0.25 | 0.26 | 0.48 |  |

**Note:** Cells are left blank where associations were not statistically significant (p > 0.01).

## **Appendix 5. Explanatory Factor Analysis Results**

The factor retention was assessed using scree plots, MAP, parallel analysis, and model fit. For DWI-Standard and ASCOT-SCT4 items, the scree plots indicated a dominant first factor, followed by evidence of a second factor before the eigenvalues began to level off. The fit indices generally favoured the 2-factor solution for the DWI-Standard and ASCOT-SCT4 items. Compared with the 1-factor user-specified model, the 2-factor solution produced a lower BIC, decreasing from −0.552 to −0.902. The incremental fit indices also improved, with RFI increasing from 0.786 to 0.833, NNFI/TLI from 0.822 to 0.871, and CFI from 0.839 to 0.895. Although the fit indices did not all indicate excellent fit, the consistent improvement across indices supported the retention of two factors.

This pattern, together with results from MAP and parallel analysis, supported examination of a two-factor solution for DWI Standard and ASCOT-SCT4 item sets. Promax-rotated factor analysis identified two correlated factors (r = 0.67). The first factor reflected broad personal wellbeing and functioning, with strong loadings from DWI items. The second factor was characterised primarily by ASCOT-SCT4 items capturing social care-related quality of life. Despite a dominant general factor, the two-factor solution provided improved model fit and conceptual interpretability.

As shown in Table A5.1, most DWI items loaded strongly on Factor 1, including meaningful life (0.80), mental health (0.72), learning (0.72), work (0.62), relationships with friends (0.59), and everyday activities (0.58). Additional DWI items with moderate loadings on this factor included physical health (0.53), respect and dignity (0.47), safety (0.40), and finances (0.41). Several DWI items also exhibited smaller loadings on Factor 2, including relationships with family (0.31), housing (0.38), and safety (0.38), indicating some overlap with domains of social care-related quality of life. Personal care remained the only DWI item with its primary loading on Factor 2 (0.37).

In contrast, the ASCOT-SCT4 items predominantly loaded on Factor 2, including food and drink (0.67), accommodation cleanliness and comfort (0.68), personal cleanliness and comfort (0.52), and safety (0.51). Some ASCOT-SCT4 items also loaded on Factor 1, notably social participation and involvement (0.51), occupation (0.52), and control over daily life (0.34), suggesting that aspects of social engagement and autonomy are also related to the broader wellbeing construct captured by Factor 1.

Overall, the pattern of loadings suggests a degree of conceptual distinction between the DWI and ASCOT-SCT4 constructs, while also highlighting areas of overlap, particularly in domains related to relationships, autonomy, and aspects of safety and living conditions.

**Figure A5.1**. Scree plot of the eigenvalues of the factors of DWI-Standard and ASCOT-SCT4

**Table. A5.1**. Oblique (Promax-rotated) factor loadings for the items of the DWI Standard and ASCOT-SCT4 (N=1328)

| **Measure** | **Item** | **Factor*** | |
| --- | --- | --- | --- |
|  |  | **1** | **2** |
| **DWI** | Relationships- Family | 0.33 | 0.31 |
|  | Relationships- Friends | 0.59 |  |
|  | Relationships- Support team | 0.41 |  |
|  | Personal care |  | 0.37 |
|  | Everyday activities | 0.58 |  |
|  | Meaningful life | 0.80 |  |
|  | Housing | 0.39 | 0.38 |
|  | Physical health | 0.53 |  |
|  | Mental health | 0.72 |  |
|  | Learning | 0.72 |  |
|  | Respect & dignity | 0.47 |  |
|  | Safety | 0.40 | 0.38 |
|  | Finances | 0.41 |  |
|  | Work | 0.62 |  |
| **ASCOT- SCT4** | Control over daily life | 0.34 |  |
|  | Personal cleanliness and comfort |  | 0.52 |
|  | Food and drink |  | 0.67 |
|  | Safety |  | 0.51 |
|  | Social participation and involvement | 0.51 |  |
|  | Occupation | 0.52 |  |
|  | Accommodation cleanliness and comfort |  | 0.68 |
|  | Dignity |  |  |

DWI, Disability Wellbeing Index; ASCOT-SCT4, Adult Social Care Outcomes Toolkit Self Complete *Only factor loadings ≥ 0.30 are presented. ASCOT-SCT4’s Dignity item was not loaded to any of the factors.

For DWI-EE and ASCOT-ER, the scree plots indicated a dominant first factor, followed by evidence of a second factor before the eigenvalues began to level off. The model fit indices indicated that the 2-factor solution provided a better fit than the user-specified 1-factor model. The BIC decreased from −0.552 to −0.902, indicating improved fit for the 2-factor solution. Incremental fit indices also improved, with RFI increasing from 0.786 to 0.833, NNFI/TLI from 0.822 to 0.871, and CFI from 0.839 to 0.895 (Table A5.2).

As shown in Table A5.2, most DWI-EE items loaded strongly on Factor 1, including everyday activities (0.80), meaningful life (0.78), learning (0.77), mental health (0.68), physical health (0.62), and personal care (0.61). Moderately strong loadings were also observed for relationships with friends (0.57), relationships with support team (0.54), work (0.53), respect and dignity (0.53), and relationships with family (0.52), with housing showing the lowest loading among DWI-EE items on this factor (0.41). Some DWI-EE items also exhibited secondary loadings on Factor 2, including housing (0.32), safety (0.43), and finances (0.38), suggesting overlap with social care–related quality-of-life domains.

In contrast, ASCOT-ER items predominantly loaded on Factor 2, including food and drink (0.78), accommodation cleanliness and comfort (0.68), personal cleanliness and comfort (0.47), control over daily life (0.44), and safety (0.49). Social participation and involvement (0.41) showed its primary loadings on Factor 1 and also displayed smaller secondary loadings on Factor 2 (0.33), indicating shared variance with broader wellbeing constructs. Dignity demonstrated the weakest ASCOT-ER loading on Factor 2 (0.34).

The factors were strongly correlated (r = 0.64), while maintaining sufficient unique variance to suggest that they capture related but distinct dimensions of quality of life. Overall, the pattern of loadings suggests a degree of conceptual differentiation between the DWI-EE and ASCOT-ER constructs, alongside notable areas of overlap, particularly in domains related to safety, living conditions, and social participation.

**Figure A5.2.** Scree plot of the eigenvalues of the factors of DWI-EE and ASCOT-ER

**Table. A5.2**. Oblique (Promax-rotated) factor loadings for the items of the DWI-EE and ASCOT-ER (N=543)

| **Measure** | **Item** | **Factor*** | |
| --- | --- | --- | --- |
|  |  | **1** | **2** |
| **DWI-EE** | Relationships- Family | 0.52 |  |
|  | Relationships- Friends | 0.57 |  |
|  | Relationships- Support team | 0.54 |  |
|  | Personal care | 0.61 |  |
|  | Everyday activities | 0.80 |  |
|  | Meaningful life | 0.78 |  |
|  | Housing | 0.41 | 0.32 |
|  | Physical health | 0.62 |  |
|  | Mental health | 0.68 |  |
|  | Learning | 0.77 |  |
|  | Respect & dignity | 0.53 |  |
|  | Safety | 0.33 | 0.43 |
|  | Finances |  | 0.38 |
|  | Work | 0.53 |  |
| **ASCOT-ER** | Control over daily life |  | 0.44 |
|  | Personal cleanliness and comfort |  | 0.47 |
|  | Food and drink |  | 0.78 |
|  | Accommodation cleanliness and comfort |  | 0.68 |
|  | Social participation and involvement | 0.41 | 0.33 |
|  | Safety |  | 0.49 |
|  | Occupation |  | 0.43 |
|  | Dignity |  | 0.34 |

DWI-EE, Disability Wellbeing Index Easy English; ASCOT-ER, Adult Social Care Outcomes Toolkit Self Complete Easy Read *Only factor loadings ≥ 0.30 are presented.

## **Appendix 6. Known-groups validity: ASCOT-SCT4/ER and DWI scores across explanatory variables**

Table A6.1 presents unadjusted descriptive comparisons of ASCOT-SCT4 and DWI-Standard and ASCOT-ER and DWI-EE scores across known-groups comparator categories derived from four variables from the SFOF questionnaire: self-rated health, meaningful leisure activities, decision-making autonomy, and self-advocacy. These variables were selected a priori because scores were expected to differ between response groups in theoretically meaningful directions.^1-4^ Scores are presented as mean ± standard deviation (SD) for each response category. These descriptive comparisons are included to aid interpretation of the adjusted regression-based known-groups analyses reported in the main manuscript.

The questions from the SFOF questionnaire and the constructed comparator groups were defined as follows:

- Self-rated health: based on the question, “Overall, do you think that your health is excellent, very good, good, fair or poor?” Responses were grouped as poor/fair, good, very good, and excellent. The poor/fair group was used as the reference category in the regression analyses.
- Meaningful leisure activities: based on the question, “Do you spend your free time doing activities that interest you?” Responses were grouped as no and yes, with no used as the reference category.
- Decision-making autonomy: based on the question, “How often do you feel you are able to have a say with the service that provides support for you?” Responses were grouped as none/a little of the time, some of the time, most of the time, and all of the time. The none/a little of the time group was used as the reference category in the regression analyses.
- Self-advocacy: based on the question, “Do you feel able to advocate (stand up) for yourself?” Responses were grouped as no and yes, with no used as the reference category.

-----------------------------------------------

Conti-Ramsden, G., Durkin, K., Mok, P. L., Toseeb, U., & Botting, N. (2016). Health, employment and relationships: Correlates of personal wellbeing in young adults with and without a history of childhood language impairment. Social Science & Medicine, 160, 20-28.

^2^ Brajša-Žganec, A., Merkaš, M., & Šverko, I. (2011). Quality of life and leisure activities: How do leisure activities contribute to subjective well-being? Social Indicators Research, 102(1), 81-91.

^3^ Tilley, E., Strnadová, I., Danker, J., Walmsley, J., & Loblinzk, J. (2020). The impact of self‐advocacy organizations on the subjective well‐being of people with intellectual disabilities: A systematic review of the literature. Journal of Applied Research in Intellectual Disabilities, 33(6), 1151-1165.

^4^ Teshale, S. M., Molton, I. R., & Jensen, M. P. (2019). Associations among decisional autonomy, fatigue, pain, and well-being in long-term physical disability. Rehabilitation Psychology, 64(3), 288.

**Table A6.1.** Known-groups validity: ASCOT-SCT4/ER and DWI scores across explanatory variables

| **Explanatory Variables** | **Groups** | **N (%)** | **ASCOT-SCT4 Mean±SD** | **DWI**  **Mean±SD** | **N (%)** | **ASCOT-ER**  **Mean±SD** | **DWI-EE**  **Mean±SD** |
| --- | --- | --- | --- | --- | --- | --- | --- |
| N |  |  | 1312 | |  | 536 | |
| Self-rated health | *Poor/Fair* | 251(19) | 0.511 ± 0.264 | 0.671 ± 0.180 | 124 (23) | 0.542 ± 0.279 | 0.688 ± 0.176 |
|  | *Good* | 380 (29) | 0.674 ± 0.210 | 0.793 ±0.128 | 169 (32) | 0.654 ± 0.235 | 0.782 ± 0.155 |
|  | *Very Good* | 387 (30) | 0.757 ± 0.170 | 0.847 ± 0.097 | 149 (28) | 0.784 ± 0.144 | 0.866 ± 0.870 |
|  | *Excellent* | 294 (22) | 0.861 ± 0.140 | 0.906 ± 0.069 | 94 (18) | 0.858 ± 0.129 | 0.914 ± 0.707 |
| *P-value* |  |  | <0.001 | <0.001 |  | <0.001 | <0.001 |
| Meaningful leisure activities | *No* | 288 (22) | 0.500 ± 0.251 | 0.691 ± 0.172 | 399 (74) | 0.511 ± 0.274 | 0.687 ± 0.190 |
|  | *Yes* | 1204 (78) | 0.768 ± 0.184 | 0.844 ± 0.116 | 137 (26) | 0.765 ± 0.184 | 0.848 ± 0.116 |
| *P-value* |  |  | <0.001 | <0.001 |  | <0.001 | <0.001 |
| Decision-making autonomy | *None/a little* | 289 (22) | 0.585 ± 0.263 | 0.747 ± 0.187 | 121 (23) | 0.540 ± 0.272 | 0.712 ± 0.192 |
|  | *Some of the time* | 287 (22) | 0.670 ± 0.228 | 0.793 ± 0.145 | 125 (23) | 0.629 ± 0.233 | 0.787 ± 0.135 |
|  | *Most of the time* | 464 (35) | 0.747 ± 0.192 | 0.832 ± 0.124 | 184 (34) | 0.761 ± 0.169 | 0.844 ± 0.124 |
|  | *All of the time* | 272 (21) | 0.818 ± 0.174 | 0.861 ± 0.103 | 106 (20) | 0.860 ± 0.151 | 0.875 ± 0.121 |
| *P-value* |  |  | <0.001 | <0.001 |  | <0.001 | <0.001 |
| Self-advocacy | *No* | 510 (39) | 0.631 ± 0.254 | 0.767 ± 0.168 | 239 (45) | 0.615 ± 0.265 | 0.756 ± 0.177 |
|  | *Yes* | 802 (61) | 0.759 ± 0.197 | 0.839 ± 0.120 | 297 (55) | 0.769 ± 0.188 | 0.849 ± 0.121 |
| *P-value* |  |  | <0.001 | <0.001 |  | <0.001 | <0.001 |

Note: P-values derived using Kruskal–Wallis tests for variables with three or more categories and Mann–Whitney U tests for binary variables.

## **Appendix 7. Comparison of demographic and disability characteristics between the excluded sample and the study sample**

Table A7 presents a comparison of demographic and disability characteristics between participants who were excluded from the analytic sample and those included in the final study sample. This comparison was undertaken to assess whether the final study sample differed systematically from the excluded sample on key observed characteristics, including age, First Nations status, language spoken, gender, and disability group.

Compared with the excluded sample, participants in the study sample were significantly older and differed in gender distribution. The study sample also had a higher proportion of participants reporting physical disability, and head injury/stroke/acquired brain injury, but a lower proportion reporting autism. There was no evidence of statistically significant differences in First Nations status, speaking English only, sensory disability, or other disability group membership.

Although some statistically significant differences were observed between the study and excluded samples, the magnitude of most differences was small to moderate, and the main characteristics of the study sample were broadly similar to the whole sample. Therefore, while some selection bias cannot be ruled out, there is no clear evidence that the exclusions materially affected the study findings.

**Table A7**.**1** Comparison of demographic and disability characteristics between the excluded sample and the study sample

|  | **Whole sample (participants who gave consent)** | | | **Study sample** | | | **Excluded Sample** | | |  |  |
| --- | --- | --- | --- | --- | --- | --- | --- | --- | --- | --- | --- |
|  | **N** | **Freq/ mean** | **Prop/ (SD)** | **N** | **Freq/**  **mean** | **Prop/ (SD)** | **N** | **Freq/**  **mean** | **Prop/**  **(SD)** | **p** |  |
| Age, Mean (SD) | 2357 | 39.2 | 18.6 | 1854 | 40.2 | 18.4 | 503 | 35.3 | 18.6 | <0.001 |  |
| First Nations (%) | 1945 | 85 | 4% | 1860 | 79 |  | 85 | 6 | 7% | 0.3238 | |
| Speaks English only (%) | 1923 | 1777 | 92% | 1838 | 1702 | 93% | 85 | 75 | 88% | 0.2244 | |
| Gender (%) | **2379** |  |  | **1871** |  |  | **508** |  |  | <0.001 | |
| Male |  | 1090 | 46% |  | 828 | 44% |  | 262 | 52% |  | |
| Female |  | 1167 | 49% |  | 942 | 50% |  | 225 | 44% |  | |
| Non-binary |  | 80 | 3% |  | 72 | 4% |  | 8 | 2% |  | |
| Prefer not to say |  | 42 | 2% |  | 29 | 2% |  | 13 | 3% |  | |
| Disability groups* | **2379** |  |  | **1871** |  |  | **508** |  |  |  | |
| Sensory |  | 782 | 33% |  | 615 | 33% |  | 167 | 33% | 0.9987 | |
| Intellectual |  | 970 | 41% |  | 745 | 40% |  | 225 | 44% | 0.0716 | |
| Physical |  | 926 | 39% |  | 761 | 41% |  | 165 | 32% | <0.001 | |
| Psychosocial |  | 1101 | 46% |  | 888 | 47% |  | 213 | 42% | 0.0258 | |
| Head Injury, Stroke, Acquired Brain Injury |  | 321 | 14% |  | 272 | 15% |  | 49 | 10% | 0.0016 | |
| Autism |  | 1051 | 44% |  | 787 | 42% |  | 264 | 48% | <0.001 | |
| Other |  | 178 | 8% |  | 140 | 8% |  | 38 | 8% | 0.9986 | |

*Percentages add up to more than 100% because participants could tick more than one option.

Continuous variables were compared using independent samples t-tests, and categorical variables were compared using chi-square tests. A p-value <0.05 was considered statistically significant.
